# Increased upconversion performance for thin film solar cells: a trimolecular composition

**DOI:** 10.1039/c5sc03215f

**Published:** 2015-10-09

**Authors:** Yuen Yap Cheng, Andrew Nattestad, Tim F. Schulze, Rowan W. MacQueen, Burkhard Fückel, Klaus Lips, Gordon G. Wallace, Tony Khoury, Maxwell J. Crossley, Timothy W. Schmidt

**Affiliations:** a School of Chemistry , UNSW , Sydney , NSW 2052 , Australia . Email: timothy.schmidt@unsw.edu.au ; Tel: +61 439 386 109; b ARC Centre of Excellence for Electromaterials Science (ACES) , Intelligent Polymer Research Institute (IPRI) , The University of Wollongong , North Wollongong , NSW 2522 , Australia; c Institute for Silicon Photovoltaics , Helmholtz-Zentrum Berlin , D-12489 , Germany; d School of Chemistry , The University of Sydney , NSW 2006 , Australia

## Abstract

Photochemical upconversion based on triplet–triplet annihilation (TTA-UC) is employed to enhance the short-circuit currents generated by two varieties of thin-film solar cells, a hydrogenated amorphous silicon (a-Si:H) solar cell and a dye-sensitized solar cell (DSC). TTA-UC is exploited to harvest transmitted sub-bandgap photons, combine their energies and re-radiate upconverted photons back towards the solar cells. In the present study we employ a dual-emitter TTA-UC system which allows for significantly improved UC quantum yields as compared to the previously used single-emitter TTA systems. In doing so we achieve record photo-current enhancement values for both the a-Si:H device and the DSC, surpassing 10^–3^ mA cm^–2^ sun^–2^ for the first time for a TTA-UC system and marking a record for upconversion-enhanced solar cells in general. We discuss pertinent challenges of the TTA-UC technology which need to be addressed in order to achieve its viable device application.

## Abstract 2

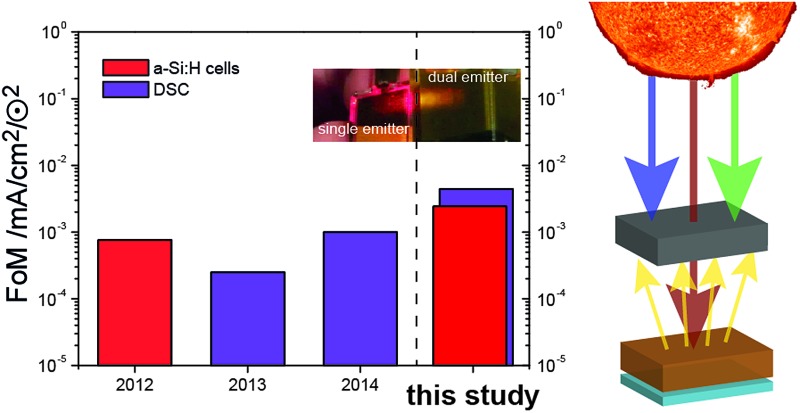
A dual-emitter upconvertor is applied to thin-film solar cells for the first time, generating record figures of merit.

## 1 Introduction

All absorbers in photovoltaic (PV) cells transmit photons with energies below their respective bandgaps, and therefore they fail to harvest the low energy portion of the solar spectrum. Photon upconversion (UC) has been recognized as a method to assist photovoltaic devices to harvest this unused sub-threshold light. The UC method can theoretically expand the utilization of the solar spectrum and thus is recognized as a potential method to exceed the Shockley–Queisser efficiency limit^1^ for PV conversion.^2–5^ A maximum solar power conversion efficiency of around 43% has been calculated for an upconversion-assisted solar cell assuming the AM1.5G solar spectrum.^3,6–8^

Essential requirements for the application of UC include a broad absorption in the sub-threshold region of the PV absorber and high UC quantum yield under incoherent low-intensity illumination. UC through sequential photon absorption (SPA) using lanthanide ion-doped materials in solid-state matrices has been studied intensively.^9,10^ However, these UC systems suffer from very weak absorption due to their Laporte-forbidden optical transitions, and very narrow atomic absorption lines.^11^ Applications of SPA-UC systems to PV devices based on gallium arsenide,^12^ crystalline silicon,^13–18^ hydrogenated amorphous silicon (a-Si:H),^19,20^ dye-sensitized solar cells (DSC)^21–23^ and organic photovoltaic materials^24^ have been demonstrated, but in consequence they mostly require relatively high solar concentration to achieve measurable current enhancement. Recently, researchers have been able to broaden the absorption range of SPA materials through the attachment of organic dyes acting as antennae,^25^ or to increase their absorption by exploiting plasmonic resonances in metallic nanostructures.^26^ However, these advanced SPA concepts still await device implementation in solar energy conversion.

In contrast, UC based on triplet–triplet annihilation (TTA-UC) involves organic molecular species, which typically have broader and stronger electronic transitions as compared to lanthanide ion-doped materials. Additionally, TTA-UC exploits the large oscillator strength of singlet–singlet transitions to absorb and emit the light, in contrast to SPA-UC, which has a weak oscillator strength to absorb and emit. Moreover, in TTA-UC, the intermediate energy storage is facilitated by long-lived triplet states of the organic chromophores (>40 μs (ref. 27)), which is important for the merging of energy from two photons arriving at different times. Consequently, TTA-UC has been proven to be an efficient photon upconversion process by various research groups^27–31^ and UC yields of greater than 30% have been measured for TTA-UC under intense monochromatic illumination.^28,32,33^ However, studies have also shown that TTA-upconversion is achievable under broad-band white-light illumination.^34–37^ Based on the promising quantum yields and the spectral tunability of TTA-UC, several applications in solar energy conversion and storage have been demonstrated ranging from solar water splitting^35,38,39^ or molecular solar thermal storage^34^ to UC-enhanced thin-film solar cells, with progressive results in the latter field being published primarily by our group.^40–45^

Despite the high UC yields shown under high illumination densities, we estimated the UC yield of our previous flagship TTA-UC system under 1 sun conditions to be just ∼1%.^46,47^ A detailed analysis based on the modeling of the TTA dynamics (see also below) allows us to identify the comparably low TTA rate of our flagship emitter species, rubrene, to be one of the dominant bottlenecks of the current system. To overcome this hindrance, we herein employ a novel dual-emitter TTA system, which indeed allows significantly higher UC quantum yields to be reached under the low-light conditions relevant to solar energy conversion. Combining the new TTA-UC system with two types of state-of-the-art thin-film solar cells we thereby obtain record current enhancements by photochemical upconversion.

## 2 Principle of TTA-UC

**Fig. 1:**
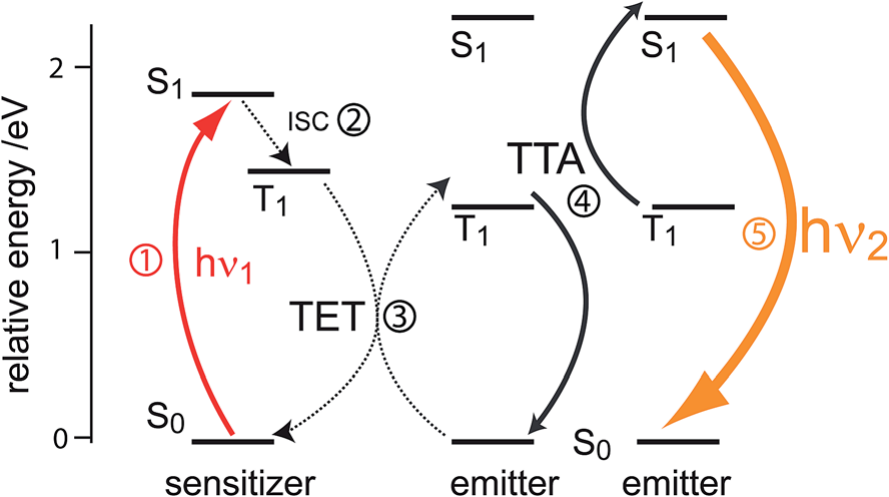
Schematic representation of photon upconversion by triplet–triplet annihilation. A ground-state sensitizer molecule absorbs a low energy photon (*hν*_1_, ), then undergoes intersystem crossing (ISC) to the first triplet state (). The energy from this triplet is then transferred *via* a (Dexter) triplet energy transfer (TET) process^48^ to a ground state emitter molecule, which populates its triplet state (). TTA occurs between two emitters in the excited triplet state *via* a collisional complex to yield one emitter in the first singlet excited state and the other in the ground state (). The excited singlet emitter emits a higher energy photon (*hν*_2_) to return to its ground state ().

Triplet–triplet annihilation upconversion is based on the co-action of two organic chromophores, a sensitizer which absorbs the incident photons and stores their energy in long-lived triplet states, and an emitter which combines the triplet energies by the TTA process. The upconversion process is depicted in Fig. 1, with the detailed mechanism given in the caption. Processes – are usually not efficiency-limiting.^49^ However, triplet–triplet annihilation itself (), is the crucial and performance-limiting step in liquid TTA-systems. Being a bimolecular process, it also gives rise to a non-linear response of the TTA-UC photon yield under low excitation intensity as triplet emitter molecules may decay by a non-radiative first-order loss channel prior to a TTA event.^27,49–51^ The dynamics of the system and the crucial role of the TTA rate has been elucidated by analysis of coupled rate equations:^2,46,49,50,52–54^
1
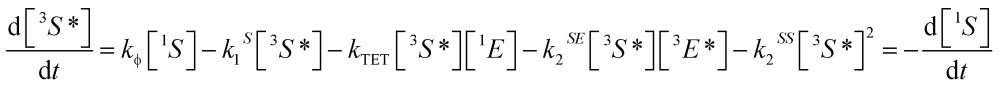

2
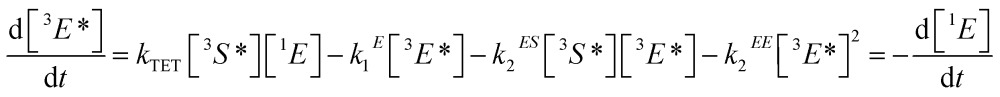



[^*n*^X] are the concentrations of the respective species, with X = E for emitters and X = S for sensitizers, with the spin states *n* = 1 for singlets and *n* = 3 for triplets (* refers to an excited species). Of the rate constants, *k*_φ_ is the sensitizer excitation rate constant brought about by absorption of photons, *k*S1 is the sensitizer triplet decay rate constant by first-order processes, *k*_TET_ is the TET rate constant between sensitizer and emitter molecules and *k*E1 is the first-order emitter triplet decay rate constant. The *k*XY2 (with X, Y = E or S) are TTA rate constants for species ^3^X* reacting with ^3^Y*. These rate equations describe the generic behavior of TTA-UC systems which has been observed and discussed in several studies.^2,46,49,50,52–54^ Here we will focus on the role of the TTA rate constant between emitters *k*EE2, being the crucial quantity for the efficacy of the TTA process.

**Fig. 2:**
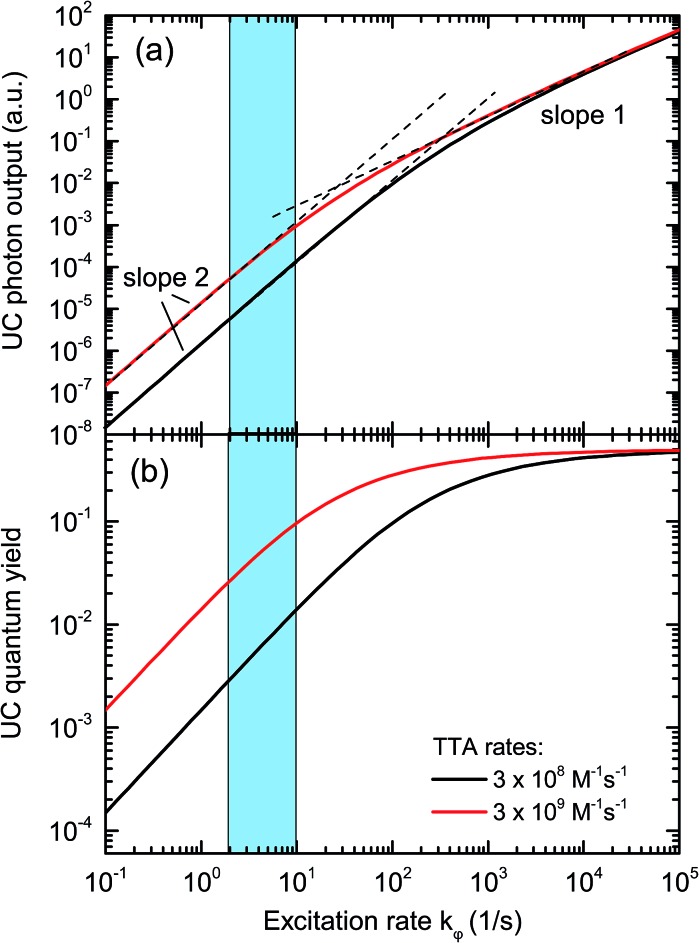
Solutions of the TTA rate equations for typical rate constants of presently employed single-emitter TTA systems (black curve), and for a 10-fold increased TTA rate (red curve). The blue area highlights the range of sensitizer excitation rate achievable under sunlight illumination. It can be seen that upon increasing the TTA rate, the UC quantum yield under 1 sun conditions is significantly enhanced.

In an experimental study employing rubrene as an emitter species, we found that the portion of emitter triplets consumed through bimolecular processes under 1 sun illumination conditions is around 1%, with the rest of the triplet molecules decaying through other processes.^47^ This corresponds to an upconversion quantum yield (QY) of only 0.5%, which limits the applicability to solar energy enhancement. One of the major factors leading to this TTA-UC bottleneck is the slow TTA rate of rubrene, which is ∼1 × 10^8^ M^–1^ s^–1^, around two orders of magnitude lower than the diffusion limit in common organic solvents.^55^ As a consequence, under low triplet concentration (*i.e.*, under low illumination), the majority of rubrene molecules in the triplet state decay back to the ground state due to the lack of opportunity to collide with another triplet. Solving the appropriate rate equations with typical values for the variables,^2^ we can see in Fig. 2 that the QY is around 1% for the excitation rates of 2–10 s^–1^ commonly realized under 1 sun illumination conditions, assuming the TTA rate constant for rubrene (black line). With increases in excitation intensity, the emitter triplet concentration increases and eventually reaches a level where the majority of triplets collide with each other, at which point the TTA process moves from a quadratic relationship with light intensity to linear one.^27,50,51,53^ By increasing the TTA rate constant by a factor of 10, the quantum yield under 1 sun conditions is increased to about 10% and the roll-over to the linear regime is shifted to lower excitation rates.

Indeed, TTA emitter materials with much higher TTA rate constants are known. For example, 2-chloro-bis-phenylethynylanthracene (2CBPEA) was found to exhibit a TTA rate constant of 5.6 × 10^9^ M^–1^ s^–1^,^52^ 50 times higher than that of rubrene.^46^ Additionally, the triplet transfer rate constant (*k*_TET_) of 2CBPEA is 5 times faster than that of rubrene with similar sensitizers.^46,52^ However, the 2CBPEA fluorescence overlaps with the Soret band absorption of the sensitizers relevant to thin-film PV devices.^40,42,44,45^ Combining 2CBPEA with these relevant sensitizers in a UC system for PV cells would therefore result in severe parasitic reabsorption of the upconverted light, rendering 2CBPEA inapplicable to solar energy conversion as a solitary emitter species.

We demonstrate here that the high TTA rate constant of a compound closely similar to 2CBPEA – BPEA in our case – can indeed be exploited by *combination* with the rubrene-based flagship TTA system. We show that a synergistic action of the two emitter species leads to a significantly increased yield of the upconverted fluorescence emitted by the rubrene species, and an accordingly increased current enhancement of a-Si:H and DSC thin-film solar cells. These two devices have absorption onsets of 1.7 eV and 1.8 eV, respectively, making them ideal candidates for UC enhancement under AM1.5G illumination.^6–8^ The combination of the dual-emitter UC system and the devices leads to record current enhancements. This UC architecture is similar to the mixed system reported by Cao *et al.*,^32^ who observed an increased quantum yield of a dual DPBF/DPA emitter system as compared to the individual components. Importantly, as the UC emission still results from rubrene, the dual-emitter system is *not* affected by parasitic absorption. We reasoned that BPEA would rather act as a triplet shuttle, which would assist in funneling triplet energy into the slowly moving rubrene molecules by means of its high TET and TTA rate constants.^49^ Details will be given in the discussion section. We begin with the description of the solar cells and the upconversion system.

## 3 Experimental

### 3.1 Solar cell preparation

Semi-transparent hydrogenated amorphous silicon (a-Si:H) p–i–n solar cells were prepared on 30 × 30 cm^2^ glass sheets by the following process sequence: 1000 nm of aluminium-doped zinc oxide (ZnO:Al) was deposited as front TCO by reactive sputtering. Then, a p-doped μc-Si/μc-SiO_*x*_/a-Si:H triple layer stack with a total thickness of 26 nm, 150 nm of undoped a-Si:H as absorber layer, and 27 nm of n-doped μc-Si were grown by plasma-enhanced chemical vapor deposition (PECVD). Finally, a 525 nm thick ZnO:Al back contact layer was sputtered. As in our previous study,^42^ the front TCO was a smooth film to achieve a sharp cutoff of the spectral response which helps the measurement of the UC effect. The increased transmittance of the newly developed p-doped layer stack^56^ allowed the i-layer thickness to be increased to 150 nm, while maintaining the peak EQE and near-infrared transmittance as in our previous studies. Using this approach, semi-transparent a-Si:H cells with 7.0% conversion efficiency were realized without any backside reflector (previously: 6.7%). For combination with the UC unit, the glass substrates were cut into 10 × 10 cm^2^ pieces, each containing 20 individual solar cells of 1 × 1 cm^2^ size.

DSC devices were produced in a manner similar to previously described.^45,57^ A dense TiO_2_ layer was deposited on clean F:SnO_2_ glass (Hartford) by spray pyrolysis, onto which a 3 μm layer porous TiO_2_ (18NR-T, Dyesol) film was screen printed. After sintering, this was placed in a dye bath containing 0.5 mM D149 (1-material) in 1 : 1 acetonitrile : *tert*-butanol. The sensitized film was sandwiched together with a platinised counter electrode (made by thermally decomposing a drop of 10 mM H_2_PtCl_6_ ethanolic solution on F:SnO_2_ glass), using a 25 μm Surlyn spacer. Electrolyte solution (0.1 M LiI, 0.6 M DMPII, 0.05 M I_2_ in methoxypropionitrile) was introduced into this cavity through a pre-drilled hole in the counter electrode, using a vacuum backfilling method. The filling port was then sealed using a small piece of Surlyn:aluminium laminate. Electrical connections were made using an ultrasonic soldering iron and Cerasolzer 186 (MBR).

### 3.2 TTA-UC solution preparation

The TTA-UC solution was prepared by dissolving {5,10,15,20-tetrakis(3,5-di-*tert*-butylphenyl)-6′-amino-7′-nitro-tetrakisquinoxa-lino[2,3-*b*′7,8-*b*′′12,13-*b*′′′17,18-*b*′′′′]porphyrinato}palladium(ii) (PQ_4_PdNA^40,58^) with rubrene (Sigma-Aldrich) and 9,10-bis-phenylethynylanthracene (BPEA, Sigma-Aldrich) in toluene to concentrations of 0.8 mM, 2 mM and 5.1 mM, respectively. The TTA-UC sample was deoxygenated through three freeze–pump–thaw cycles using liquid nitrogen cooling, during which the solution was pumped down to the order of 10^–3^ mbar in a custom vacuum cuvette. A concentration of 5.1 mM of BPEA was chosen as it is close to its solubility limit in toluene and does not lead to recrystallization during freeze–pump–thaw cycles. Initial investigations revealed that a 3 : 1 ratio of BPEA : rubrene provided the most significant increase to UC intensity.

**Fig. 3:**
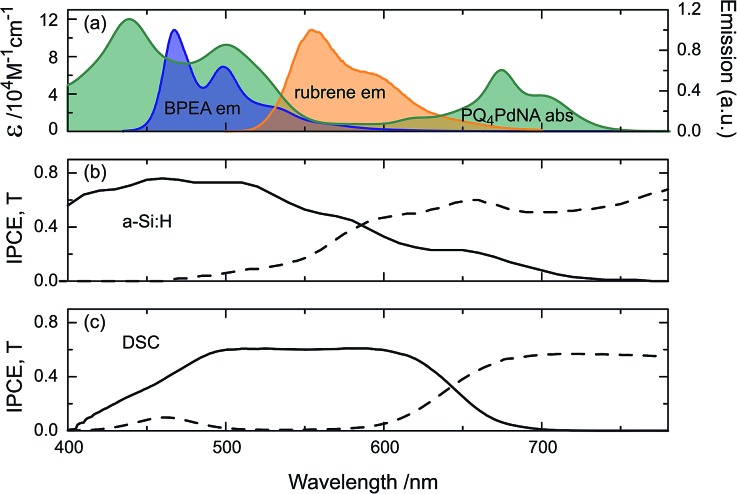
The spectral properties of the two UC/device assembles in this study: (a) absorption of PQ_4_PdNA (green) and emission of rubrene (orange) and BPEA (blue). IPCE (solid) and transmittance (dash) of (b) a-Si:H, illuminated through glass substrate and (c) DSC, illuminated through working-electrode.

Fig. 3 shows the absorption spectrum of PQ_4_PdNA, emission spectra of rubrene and BPEA, as well as the IPCE and transmission curves of the two solar cells. It is clear that the sensitizer is readily able to harvest light transmitted by both devices. Although containing two emitter species, the UC solution emits exclusively at the wavelength of the lower-energy emitter S_1_ state.^32^ This way, parasitic absorption is avoided as rubrene emits within the absorption window of the sensitizer and the emission spectrum matches well with the a-Si:H and DSC spectral responses.

### 3.3 Optical coupling

In previous studies we have optimized the combined optics of solar cell/UC unit assemblies.^40,41,43^ We found that the UC performance is optimal if the UC material is combined with a back reflector and its thickness chosen such that the reflector is positioned at approximately the characteristic absorption length (1/*e* decay) of the incident light at the sensitizer peak absorption.^40^ After propagating through the TTA-UC medium twice, the light at the peak absorbance of PQ_4_PdNA will then attenuate to 1/*e* ^2^ of its original intensity. This means that approximately 13% of the incident light leaves the UC medium, but the resulting effective concentration of the incident light leads to a net increase of UC photon yield due to the nonlinear response of the UC unit.^43^ For typically achievable sensitizer concentrations the optimum thickness is in the 100 μm range. To realize such thin effective thicknesses of the UC medium, we add silver-coated glass spheres with 100 μm diameter to the 1 cm diameter cuvette. The closely packed spheres create cavities of appropriate size in which the UC medium resides and thus help to efficiently outcouple the upconverted light.^41^ The concentration of PQ_4_PdNA was optimized for the cavity size created by the 100 μm silver-coated spheres. The front of the 1 cm cuvette with the degassed TTA-UC sample was optically coupled to the back of the a-Si:H (ZnO:Al) and DSC (working-electrode) by means of immersion oil (Sigma-Aldrich, *n*_D_^20^ = 1.516).

### 3.4 Measurement and data analysis

The current enhancement of solar cell devices brought about by TTA-UC is measured using a pump–probe technique.^40–45^ Since TTA-UC is a non-linear process under low excitation photon flux^27,50,51,53^ (Fig. 2), the low-intensity monochromated probe beam used to measure the incident photon-to-current efficiency (IPCE) in common measurement setups alone will not attain a significant TTA-UC effect. To yield a measurable UC response we therefore employ a continuous wave (CW) bias light in the form of a 670 nm diode laser, selectively exciting the sensitizer. The pump beam excites the TTA-UC solution behind the PV sample to provide a background concentration of emitter triplets to increase the upconverted photon yield induced by the chopped probe light, allowing comparisons to be made on the basis of excitation rates exerted by the pump beam. The monochromatic probe beam was chopped and the resulting signal (from the device) was recorded by lock-in amplification. The chopping frequencies used were 117 Hz and 23 Hz for a-Si:H and DSC respectively.

The analysis of our IPCE data relies on the comparison of IPCE curves taken with and without the UC effect. It turns out that the measurement without the UC effect is a non-trivial task. We cannot physically remove the UC unit as this will alter the optics of the semitransparent solar cell device and impede a direct comparison of the IPCEs. However, the UC response without the bias beam is negligible, and we therefore take this situation to be the baseline IPCE. The probe energy is ∼1 order of magnitude weaker than the lowest pump intensity employed in this study and therefore the UC intensity will be ∼100× weaker.^41^ Furthermore, this approach will result in an underestimation of the UC-derived current enhancement. A second issue concerns switching off the bias beam: although the bias laser energy is below the nominal bandgap of the PV absorbers employed here, their absorption tails may still absorb the bias beam. Even though an eventual DC current contribution from the bias will be filtered out by the lock-in detection technique, if the cell has a nonlinear response, artifacts might still be induced. We therefore do not turn off the bias beam, but laterally displace it on the solar cell area such that the probe beam is probing an unbiased region of the UC unit. By misaligning the pump and probe beams, TTA-UC generated by the probe beam is minimized while an eventual weak current-bias from the UC induced by the pump is maintained.

After measuring an IPCE response curve of a device under monochromatic illumination from 500 nm to 780 nm with the pump and probe beam aligned, we repeated this measurement with the pump and probe beam misaligned. For both the a-Si:H and DSC devices, 6 sets of aligned and misaligned IPCE measurements were taken and averaged. The pump intensity was then adjusted in order to probe the UC effect at different excitation powers. Further detail on the measurement procedure can be found elsewhere.^45^

The IPCE traces with UC contribution are divided by the corresponding baseline IPCE measurements to obtain IPCE enhancement curves. We found earlier that the resulting enhancement traces can be understood and modeled, taking into account the solar cell transmission and sensitizer absorption,^41^ confirming the enhancement to be TTA-UC related. Furthermore, integrating the measured enhancement curves over the AM1.5G spectrum, the enhancement of the solar cell photocurrent (Δ*J*_SC_, in mA cm^–2^) under the given solar concentration factor defined by the pump beam intensity can be determined.

In order to quantify the effective solar concentration sensed by the UC unit, we calculate the rate of excitation of an individual sensitizer molecule *k*_φb_ = *σ*(*λ*_b_)*T*_SC_(*λ*_b_)*I*_b_, with the irradiation *I*_b_ of the bias beam in photons per area per time and the bias laser wavelength *λ*_b_. We then compare this rate to the excitation rate brought about by the AM1.5 solar spectrum filtered by the solar cell transmission (*k*_φ_). *k*_φ_ is calculated by multiplication of the AM1.5G solar spectrum, *ρ*_⊙_, in photons cm^–2^ s^–1^ nm^–1^ by the transmission of the solar cell, *T*_SC_, and integrating the product of this with the absorption cross section of the sensitizer species, *σ*(*λ*) in cm^2^,

3 *k*_φ⊙_ = ∫*ρ*_⊙_(*λ*)*T*_SC_(*λ*)*σ*(*λ*)d*λ*.

Typical values for *k*_φ⊙_ are in the 2–10 s^–1^ range and depend on the solar cell transmission. The ratio *C* = *k*_φb_/*k*_φ⊙_ then gives the effective solar concentration sensed by the upconverter. Since the two devices have different transmittances of the solar spectrum, the excitation rates from the solar spectrum are not identical even with the same TTA-UC materials and measurement conditions. As a consequence, the two devices were studied under different solar concentration ranges. Δ*J*_SC_ values were normalized by dividing by the square of the concentration factor (*C* ^2^) to account for the inherently quadratic response of the TTA-UC process for low illumination densities (Fig. 2).^40–45,59^ The normalized Δ*J*_SC_ values are our figures of merit (FoMs) for comparisons between UC/device pairs, and equal the current enhancement by UC that would be measured under 1 sun conditions. Further details of experimental techniques, data analysis and modeling can be found in our recent publications.^40–42,45^

## 4 Experimental results

**Fig. 4:**
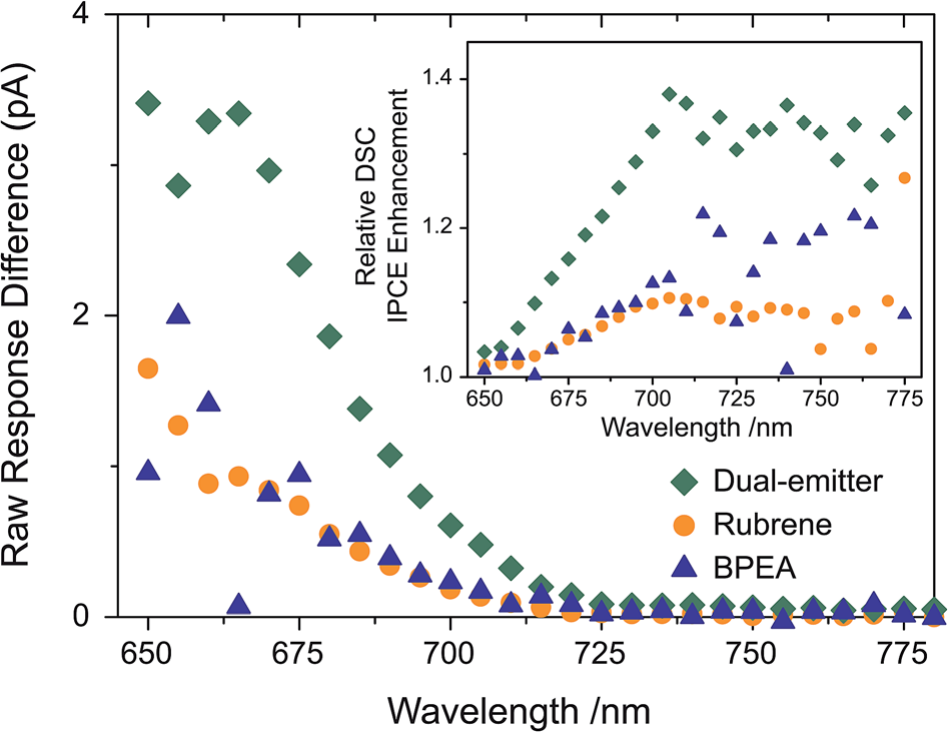
Extra current generated from a DSC due to TTA-UC with dual-emitter (green), rubrene only (orange) and BPEA only (blue) UC system. The inset shows the IPCE enhancement of the DSC with the three different UC systems.

The initial experiment in this series started by studying the behaviour of the TTA-UC solutions coupled to a DSC upon changing the emitter composition. Firstly, IPCE control measurements were established with a rubrene only, a BPEA only and a dual-emitter (BPEA : rubrene = 3 : 1) UC system with the same *total* emitter concentration each, and in combination with a DSC. The short circuit current responses without UC contribution were subtracted from the responses with activated UC unit and the resulting raw current enhancement traces are shown in Fig. 4, which represent the extra solar cell current generated due to the presence of the different TTA-UC solutions. Despite the parasitic absorption of the BPEA emission by the sensitizer, its higher TTA rate constant makes up for this, and the increased currents of BPEA and rubrene alone are near identical. We saw that in the presence of both emitter species, ∼3 times more UC-related current was generated by the DSC, as compared to the situation where only a single emitter (either rubrene or BPEA) was employed. It is important to note that this occurs despite the total emitter concentration remaining constant, and points towards a synergistic action of rubrene and BPEA in the utilization of the sensitizer triplet density.^49^

**Fig. 5:**
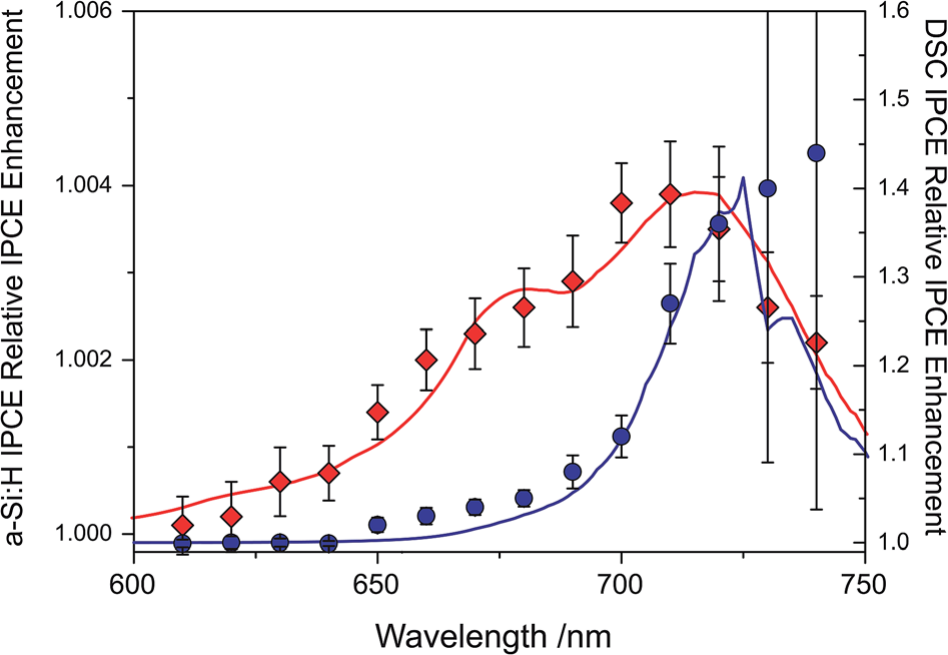
Relative enhancement trace for a-Si:H device (, red trace) tested with ∼1.4 suns equivalent illumination (*λ* = 670 ± 5 nm) and a DSC (∘, blue trace) tested with ∼1.3 ⊙ equivalent illumination, including the modeled traces for the two devices.^40,41^

The IPCE enhancements of the two UC/device assemblies, obtained by dividing UC-assisted and baseline IPCE, are shown in Fig. 5 at an effective solar concentration for the a-Si:H cell of 1.4 ⊙ and for the DSC of 1.3 ⊙. The error bars on the traces are the standard deviations from point averaging at the respective wavelength. Since the DSC has a significantly lower IPCE in the range of 680 < *λ* < 750 nm, it has a much more pronounced relative IPCE enhancement compared to the a-Si:H device. This shows that direct comparisons of UC/device assemblies drawn from the relative enhancements are not sensible.

**Fig. 6:**
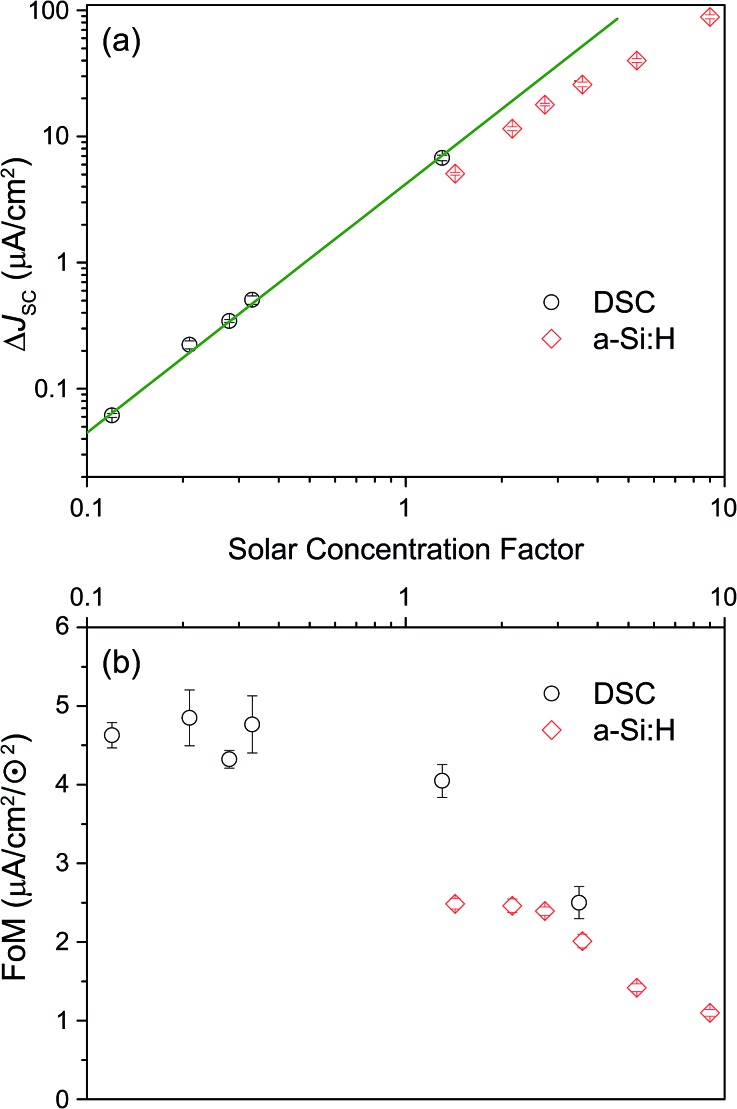
(a) Dependence of calculated current gain (Δ*J*_SC_) on effective solar concentration (both axes on a logarithmic scale) for the a-Si:H device () and DSC (∘). (b) Figure of merit (FoM) as a function of solar concentration for a-Si:H () and DSC (∘).

The IPCE measurements and the determination of the short-circuit current increase Δ*J*_SC_ as well as of the FoM were repeated as described above for a range of different effective solar concentrations (0.1 to 9 ⊙). The results are shown in Fig. 6. Panel (a) reproduces the generic behavior of TTA-UC systems shown in Fig. 2 by displaying a quadratic response of the UC-related current for low excitation densities which turns into a sub-quadratic increase above ≈3–5 ⊙. This behavior was also seen experimentally in many studies analyzing the upconverted fluorescence intensity upon varying the pump intensity.^2,5,50,51,60,61^ The fact that Δ*J*_SC_ (⊙) is sub-quadratic already beyond 3–5 ⊙ indicates that the TTA efficiency of the UC system is beginning to saturate.^27^ The comparison to the simulated QY from Fig. 2 suggests that our dual-emitter UC system is indeed operating at a higher effective TTA rate than that of rubrene: the changeover to the sub-quadratic regime should not happen below ≈25 ⊙ for a pure rubrene system (Fig. 2 taking into account that *k*_φ_ at 1 sun is 4 s^–1^ for PQ_4_PdNA). The position of the changeover at ≈3–5 ⊙ suggests a roughly 10-fold increased effective TTA rate. Although further studies are needed to substantiate this claim, we take it as a strong indication that BPEA increases the effective TTA rate of rubrene-based TTA systems.

As seen in Fig. 6b the FoM values are constant in the quadratic regime from panel (a) and decay to lower values for higher concentration factors. The FoMs can be used for meaningful comparisons between different UC/device assemblies from both the present report and previous studies. The DSC FoM was ∼4.5(5) × 10^–3^ mA cm^–2^ ⊙^–2^, deviating from quadratic at illumination levels of 3 ⊙, while the a-Si:H device displayed an FoM of ∼2.4 (0.1) × 10^–3^ mA cm^–2^ ⊙^–2^ up to 3 ⊙.

Apart from the impact of the sub-quadratic response for the highest excitation densities, it seems that the DSC device outperforms the a-Si:H device also in regions where the illumination density is moderate.

Inspecting the baseline IPCE spectra in Fig. 3, it appears that the DSC can make better use of the upconverted light as it displays a constant IPCE of roughly 0.6 across the entire region of the rubrene emission, while the a-Si:H cell IPCE is reduced for *λ* > 500 nm. The advantage of the DSC over the a-Si:H device is that its spectral response can be readily tuned by choosing a specific dye to match the UC emission. For example, the D149 dye employed here is chosen for the DSC to provide a good spectral response to rubrene emission. The a-Si:H device on the other hand has higher transmission in the (infra)red region and results in enhancements across a broader spectral range (Fig. 5). Nonetheless, this fact does not compensate for the mismatch of a-Si:H IPCE and rubrene emission.

## 5 Discussion

### 5.1 Increase of UC quantum yield by dual-emitter system

There are, to our knowledge, two studies that have shown a beneficial effect of combining two emitter species with a single sensitizer. Cao *et al.* combined 1,3-diphenylisobenzofuran (DPBF) and 9,10-diphenylanthracene (DPA) as emitters with platinum(ii) octaethylporphyrin (PtOEP) as sensitizer and observed a significantly enhanced UC quantum yield with UC emission primarily from the lower-energy emitter S_1_ state (*i.e.*, from DPA). They attributed the beneficial effect to hetero-TTA between DPA and DPBF and could also demonstrate synergistic behavior from a triple-emitter system with DPA/DPBF and anthracene.^32^ Conversely, Turshatov *et al.* only saw significant enhancement in a two emitter system when the two emitter species were chemically bonded together.^62^ However, Turshatov *et al.* prepared unusual TTA-UC samples in that the emitter with the lower first singlet excited state (the final emitting species, E2) had a higher triplet energy level than that of the sensitizer and the other emitter, E1, leading to a complicated triplet energy transfer (TET) process. In addition, the emitter concentrations prepared by Turshatov *et al.* were on the order of 10^–5^ M, two orders in magnitude lower than those prepared by Cao *et al.* and other efficient TTA-UC systems.^27,50^

Only rubrene emission is seen in our dual-emitter system, and this can occur under several different circumstances: there can be annihilation occurring between BPEA triplets, with subsequent resonant energy transfer to nearby rubrene species; there can be hetero-annihilation resulting only in singlet excited rubrene; and there can be dominantly homo-annihilation between triplet rubrenes. The concentration of rubrene is 2 mM, which would bring about a quenching rate for the BPEA triplet state of several × 10^5^ s^–1^. The timescale for this process is in the several microsecond range, a small fraction of the BPEA triplet lifetime. As such, it is likely that in this case the BPEA triplets are largely quenched by rubrene, and that the rubrene triplet concentration under operating conditions is much higher than that of BPEA triplets. The dominant TTA mechanism is thus possibly rubrene homo-annihilation. However, hetero-annihilation events are likely to be not insignificant on account of the faster diffusion of BPEA. If the quenching of BPEA triplets by rubrene is only 90% efficient, hetero-TTA events could still contribute substantially, due to the order of magnitude higher hetero-TTA rate.^49^ However, there is another benefit of having a dual-emitter UC system: reduction of self-quenching and self-absorption of emitter emission. Since BPEA rapidly quenches the sensitizer triplets, the concentration of rubrene can be reduced as it is no longer serving as the primary triplet harvester. The consequence is that after TTA, the emission will have less reabsorption due to the overlap in the absorption and emission spectra. The detailed kinetics of the dual emitter system will be reported in a future study.

### 5.2 Prospects of UC-enhanced solar cells

**Fig. 7:**
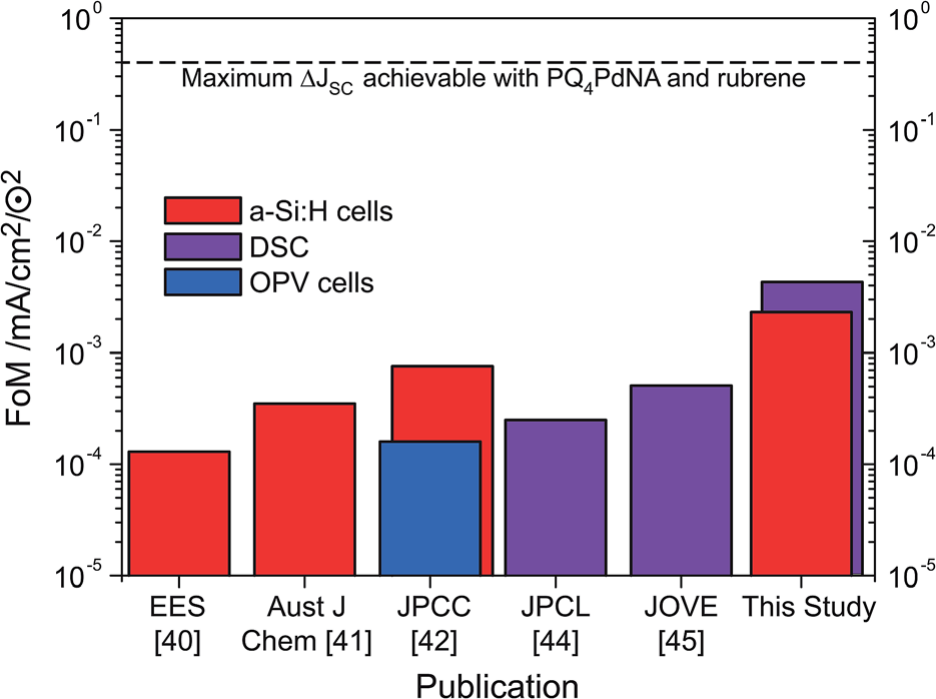
The evolution of the FoM applied to a-Si:H cells (red), DSC (purple) and OPV (blue) in logarithmic scale.

Through judicious selection of UC materials, solar cells and the optics of the combined system as well as by an improved upconvertor formulation, the current enhancement under sunlight conditions (*i.e.*, FoM) has steadily increased over the last three years (Fig. 7). With the dual-emitter TTA-UC system presented herein, new FoM records for both a-Si:H and DSC devices have been set, exceeding the 10^–3^ mA cm^–2^ ⊙^–2^ benchmark for the first time. The FoM has been improved by a factor of 40 since our first report in 2012,^40^ and – to our knowledge – now marks the record current enhancement for any upconversion-assisted solar cell, also surpassing the latest results for crystalline silicon solar cells enhanced by lanthanide UC of 1.92 × 10^–3^ mA cm^–2^ ⊙^–2^.^63^ Although the progress in TTA-UC device application is significant and the current results with dual-emitter TTA systems are promising, the obtained current enhancement under 1 sun conditions still lags behind the value of 0.4 mA cm^–2^ ⊙^–2^ which we estimated to be the absolute upper limit of PQ_4_PdNA/rubrene TTA-UC systems based on detailed optical calculations.^42^

The question, therefore, is how to further increase the quantum yield of TTA-UC under low-illumination conditions. A possible guideline for this task is given in the form of the steady-state solution of the rate eqn (1) and (2), assuming inefficient TTA-UC:4
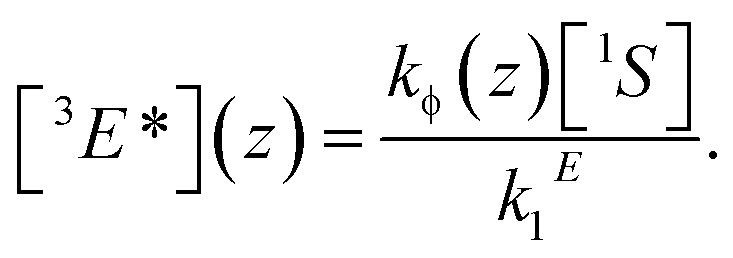



In order to increase the UC yield by increasing the steady-state concentration of emitter molecules in their triplet excited state [^3^E*], one has to lengthen the triplet lifetime 1/*k*E1, increase the concentration of sensitizers [^1^S] and its excitation rate *k*_φ_.

The triplet lifetime of the emitter is governed by its rate of reverse intersystem crossing, and its tailored extension requires molecular engineering of the emitter species. To this end, the vibrational spectrum of the molecule is key as it assists the spin-forbidden intersystem crossing. UC enhancement of 20% has been found from the deuteration of perylene from a red to blue UC system.^64^ This demonstrates that UC efficiency can be improved by phonon-engineering. However, modifications to the molecular structures of commercially available dyes may increase the cost of an upconversion system significantly.

To increase the excitation rate of the sensitizers, one can either (i) increase the incident photon flux or, (ii) introduce near-field enhancement around the sensitizer species. Due to the inherent nonlinearity of the UC response with respect to incident photon flux, any focussing of the incident light leads to an increase of UC efficiency. This effect is already utilized by the presence of a back reflector, and can be exploited much further: we recently introduced a focusing microstructured back reflector into a TTA-UC system, with the aim to increase the local light intensity and thus triplet concentration. Doing so we acquired a 20% larger Δ*J*_SC_ from an a-Si:H device as compared to using a flat mirror under the same conditions.^43^ Another viable approach to micro-optical enhancement would be to embed a TTA-UC film into a Bragg reflector, which changes the density of photon state of the emitter and may lead to emission enhancement by near-field effects.^65^ Alternatively, the excitation rate of the sensitizer and even the emission rate of the emitter may be enhanced by the presence of plasmonic resonances locally increasing the electric field.^66,67^ The TTA-UC enhancement by plasmonic effect has been realized by Poorkazem *et al.*,^68^ Baluschev *et al.* ^69^ and Xian *et al.* ^70^ with significant increase in TTA-UC achieved.

A third, equally important lever to higher UC yield is the concentration of the active species. Liquid systems are restrained to the mM range due to limited solubility of, primarily, the sensitizer species. For this reason, solid-state approaches are a very active field of TTA-related materials research,^52,71–79^ and might also allow for easier device integration and encapsulation as compared to the liquid systems. The interested reader is referred to the review article of Simon and Weder.^80^ Most approaches presented so-far rely on the blending of the active species into a solid polymeric host, and mostly suffer from aggregation and phase separation when the dye load is increased. Thus far the most efficient TTA systems still reside in the liquid phase, and new architectures are required to increase dye load while preventing aggregation and proximity self-quenching. An elegant approach would be covalently linking sensitizer and emitter species.^81^ Recent studies imply that this indeed increases the UC yield in liquid solutions,^82^ while earlier studies have found an increase of the TTA-UC photon yield of a porphyrin sensitizer end-capped solid TTA emitter^83^ as compared to a porphyrin-doped host. Although others have argued that the gain in UC efficiency in this system was minute and relate this finding to a possible exciton back-diffusion,^80^ these strategies point in the right direction. The density of active species might also be increased by immobilizing them on nanoparticles.^84,85^ This strategy is particularly promising regarding the sensitizers as their solubility is usually the limiting factor and their surface tethering might assist in avoiding unwanted self-TTA between them. Moreover, a matrix-free TTA-UC system has been recently demonstrated which does not require de-oxygenation for efficient UC to take place, and likewise allows higher concentrations of the active species.^86^ In summary, there are still many options unexplored regarding the advanced design of TTA-UC systems for solar energy conversion. Combining the different strategies outlined above might ultimately allow exploiting the full current enhancement potential of TTA-UC and pave the way to its commercial application.

## 6 Conclusion

The present study demonstrates a new benchmark for upconversion-assisted solar cells regarding the photocurrent enhancement by applying a dual-emitter triplet–triplet annihilation upconversion system to a-Si:H and dye-sensitized solar cells. The maximum photocurrent enhancements under AM1.5 conditions are 2.4(1) × 10^–3^ mA cm^–2^ ⊙^–2^ for the a-Si:H cell and 4.5(5) × 10^–3^ mA cm^–2^ ⊙^–2^ for the DSC devices, and represent a significant improvement step as compared to previous results. The result was accomplished through a TTA-UC system incorporating a second emitter species which assists the classical TTA-UC couple by enhancing the effective TTA rate while keeping UC emission in the desired wavelength window with minimal parasitic reabsorption. The exact nature of the contribution of this material being the subject of future fundamental studies, we thus highlight the importance of multicomponent TTA systems. The photocurrent enhancement is expected to advance further with optimizations of the new TTA-UC system, as well as semitransparent thin-film solar cell architectures, highlighting the promising nature of TTA-UC for application in thin-film photovoltaic devices as well as for solar water splitting.^35,38^
